# Guiding principles of AI: application in animal husbandry and other considerations

**DOI:** 10.1093/af/vfae045

**Published:** 2025-01-04

**Authors:** Andrea Rosati

**Affiliations:** EAAP—The European Federation of Animal Science, Rome, Italy

**Keywords:** AI, animal husbandry, considerations, guiding, principles

**Implications**

Increased efficiency and productivity: AI can significantly enhance decision-making in various aspects of farm management, from feeding to animal health monitoring. Real-time data analysis enables resource optimization, leading to reduced waste and improved efficiency.

Animal welfare improvement: AI tools can monitor animals’ health and welfare in real-time, predicting potential issues before they arise, allowing for earlier interventions to ensure better animal care.

Sustainability: AI technologies can help reduce environmental impact by optimizing the use of resources like water, feed, and energy, contributing to more sustainable farming practices.

Shift in competencies: AI requires specialized knowledge to operate, leading to a shift in required skill sets. Farmers and agricultural workers will need to develop new technical skills to manage AI technologies effectively.

Challenges of data availability: AI’s potential is often hindered by the lack of high-quality, diverse datasets. The success of AI models depends on the availability of comprehensive data covering different species, environments, and farm conditions.

Ethical and privacy concerns: The integration of AI into farming raises issues regarding data security and privacy. Sharing detailed farm data with AI providers may expose sensitive business information, creating potential risks.

Impact on decision-making: AI may surpass human intuition in making long-term predictions, leading to a shift where AI, rather than farmers, could drive most operational decisions. This raises concerns about the loss of human control in farm management.

Disparities in global adoption: AI technology will develop unevenly across different regions due to varying economic and technological capabilities, potentially widening the gap between advanced and less developed agricultural sectors.

Governance and control of AI: The control of AI development and deployment is concentrated in a few multinational corporations, raising concerns about dependency on these entities and their geopolitical influence over critical sectors like livestock farming.

Trust in AI: Relying on AI for critical decisions, especially when outcomes may seem counterintuitive initially, challenges the trust farmers place in these technologies. This issue will become increasingly critical as AI continues to evolve and influence farm management.

## Introduction

Some argue that the rise of Artificial Intelligence (AI) is charting a disquieting course for the future, while others are convinced that it will lead to a much better tomorrow. One thing, however, remains undeniable: although no one can accurately predict where this technological revolution will take us, it is clear that it will profoundly transform our lives, both professionally and personally. We might consider it a continuation, with an immense evolutionary leap, of the scientific discoveries that shaped the industrial era. Yet at the same time, it represents something entirely novel, a form of intelligence capable of peering far beyond the limits of our vision.

## From Advanced Data Analysis to Deep Learning

In livestock farming, the use of AI has so far mainly focused on advanced data analysis rather than the implementation of deep learning. At present, most applications use data from multiple sensors, which are integrated into statistical and analytical models to provide valuable insights through automated processes. However, it is evident that deep learning will soon take on a more prominent role as the technology evolves, finding its way into livestock farming operations (De Oliveira et al., 2023). It is essential to clarify a common misconception: neural networks and deep learning, while related, are not the same. Neural networks are computational models inspired by the workings of the human brain. They consist of nodes, called neurons, arranged in successive layers. Each neuron receives inputs, applies a mathematical transformation, and produces an output that can be passed to subsequent layers. Traditional neural networks usually have few layers: an input layer, one or two hidden layers, and an output layer. These networks have been used effectively to tackle classification, regression, and other machine learning problems. Deep learning, on the other hand, is a branch of neural networks, specifically of the deep kind (hence the term “deep”). What sets it apart is the presence of many hidden layers, which can reach tens or even hundreds of levels. As the number of layers increases, models become more complex and able to learn more detailed and sophisticated representations.

## AI Application in Livestock Farming

The evolution of AI is extraordinarily rapid and ever-accelerating. What seems cutting-edge today may be outdated within months. Therefore, it is crucial to stay constantly updated. This process will be anything but simple, and it will require highly specialized professionals to assist companies in managing AI applications. AI functions will undoubtedly be multifaceted, not limited to a single service but capable of orchestrating the entire management of a livestock farm. Data will be collected by an advanced Internet of Things (IoT), with information coming from both inside and outside the farm, gathered through various methods and units of measurement (Neethirajan, 2023). Consequently, AI will make decisions on every aspect of farm management, from identifying replacement animals to feed choices and the management of sales and purchases. The decisions currently made by farmers will increasingly be delegated to or suggested by AI. The quality of the incoming information is crucial: the more accurate the data, the better the results and the efficiency of the farm. AI solutions will need to be highly flexible, adaptable to the different structures and management styles of livestock farms, while ensuring economic efficiency in the production of AI systems. These solutions must be specifically “trained” on the farm before they can be operational. AI opens up new opportunities to optimize operations, improve animal welfare (Papakonstantinou, 2024), and increase productivity along the entire supply chain by leveraging technologies such as precision farming and predictive analytics (Fuentes et al, 2022). However, the widespread adoption of AI models is currently hindered by a lack of high-quality datasets. A significant obstacle is access to a sufficient amount of diverse data, which is essential for effectively training AI algorithms. These datasets must include information across different species and breeds, as well as varied environmental conditions and management practices, to ensure that AI is generalizable and not limited to the contexts in which it was initially trained. AI can improve various aspects of livestock management, including feeding, disease prevention, genetic selection programs, and resource allocation. By utilizing real-time data streams from IoT sensors, satellite images, and remote sensing technologies, AI can optimize resource use, reduce waste, and minimize environmental impact. AI algorithms already provide valuable insights into animal welfare, underscoring their transformative potential in livestock management. This progress represents a true paradigm shift, paving the way for a more sustainable and efficient future for livestock farming. The main goal of AI in livestock farms should be to enhance efficiency through optimal decision-making in every aspect of farm management. In practice, AI acts as a constant consultant, supporting complex decisions, minimizing errors, and suggesting the best solutions for each situation and objective. We should consider AI as a resource from which to continually demand more, so that it can provide increasingly precise and accurate answers.

## How AI Addresses Production and Sustainability Challenges

AI offers a revolutionary solution to address the main production and sustainability challenges in animal husbandry. A central issue is management inefficiency, which leads to resource wastage, including feed, water, and energy. Through the analysis of data collected from sensors and IoT devices, AI enables real-time monitoring of animal and environmental conditions, optimizing resource use. For example, advanced algorithms can automatically adjust feeding based on each animal’s specific needs, reducing waste and improving efficiency.

From a sustainability perspective, AI helps reduce environmental impact by collecting and analyzing data related to land use, greenhouse gas emissions, and energy consumption. AI supports decisions that reduce the ecological footprint and make the entire production system more resilient, predicting environmental or climatic stress scenarios and enabling farmers to take timely action. Disease prevention is another area where AI can have a significant impact. By monitoring animals’ vital parameters, AI can detect anomalies early, preventing potential outbreaks and reducing the use of preventive drugs. Additionally, AI can optimize genetic selection programs, improving productivity and resilience through precise analysis of genetic traits.

In summary, AI is a key ally in increasing efficiency, reducing environmental impact, and improving animal health and welfare, contributing to a more sustainable and competitive livestock industry.

## Future Developments in Livestock Farming

The future of AI-assisted livestock farming is difficult to predict, as it requires imagining how these technologies will evolve and adapt to the needs of farms. Beyond improving the services already mentioned, several future scenarios can be envisaged. For instance, farm management automation could become a reality through AI, which would be capable of coordinating activities such as staff management, logistics, and maintenance. Another potential development is advanced predictive analytics, which would allow farmers to anticipate health and behavioral problems in animals, optimizing their feeding and reproduction accordingly. Personalized feed management, based on the specific needs of each animal, could also become one of AI’s concrete applications. AI could also help optimize natural resource use, such as water and energy, reducing operating costs and environmental impact. Furthermore, advanced genetic selection techniques could be employed to accelerate livestock improvement. Lastly, smarter and more transparent supply chains would enable faster responses to market demands, increasing farms’ competitiveness. In conclusion, AI promises to make livestock farms smarter, more sustainable, and competitive, enhancing management efficiency and responsiveness.

Despite its numerous advantages, the adoption of AI in livestock farming presents certain challenges. Data security and privacy are major concerns, as are the technological infrastructures needed, which can be complex. The reliability of the sensors used may not always be guaranteed, leading to problems with data accuracy. Another difficulty lies in correctly interpreting the data collected, which requires specialized skills. Ethical implications must also be considered, alongside the high costs of implementing new technologies. Finally, environmental challenges and a lack of awareness about AI pose additional obstacles. Thus, while AI represents a revolution in the livestock sector, its adoption still requires significant efforts in terms of technology, infrastructure, and adequate training.

## Human vs. AI

When a farmer faces a complex issue or forecast, which is always a challenge, they try to explore every possibility, from the most plausible to the least likely. It is akin to someone observing a chessboard, attempting to anticipate every possible future move, as if every current configuration contained the entire range of subsequent scenarios. AI operates similarly in its systematic way: whether playing chess, setting up a search, or managing a business, AI relies on pure computational power, acting like an unstoppable force. In contrast, humans, though limited in their predictive abilities, use less linear methods. Up to this point, we have defended humanity by highlighting how, thanks to intuition, we can sometimes match machine efficiency. But how long will this balance last? Considering the evolutionary pace of technology compared to that of human intelligence, the outcome seems inevitable. While farmers base their decisions on memory, experience, abstraction skills, and intuition, AI operates differently: it doesn’t reflect or intuit; it relies on algorithms, using only computational power, making decisions based on a system of pre-organized rules and past observed experiences with feedback.

Each time there is a different possibility—essentially, with each slight variation in initial conditions, AI generates a new decision tree. AI explores every branch of this tree and selects the one leading to the most advantageous result. This ability to analyze infinite possibilities makes AI an unsurpassable force when it comes to long-term predictions, often leaving human intuition far behind.

In a constantly changing context like livestock farming (with different animals, varying diets, changing weather conditions, different production levels, etc.), each new scenario creates a new decision tree. With enough computational power, AI can project itself into the future, accurately predicting situations that remain unclear to the farmer until they occur.

Farm management has traditionally relied not solely on calculation, but on a blend of experience, intuition, and human decision-making. This is because livestock decisions are often too intricate, with too many interconnected factors, to be handled with a purely computational approach. However, with the advancement of AI, what was once considered impossible to compute is now becoming manageable, and AI is beginning to show that, in the long term, its ability to analyze and predict may even surpass the most refined human intuition.

In the face of this evolution, the inevitable question arises: to what extent can humans still compete? Is it only a matter of time before machines, with their exponential learning and evolving capabilities, eclipse human intuition, relegating farmers to the role of supervisors rather than decision-makers? (Liao, 2020)

When it comes to scientific research in livestock farming, the impact of AI is still difficult to imagine. Scientific research in livestock has often been helped by discoveries made in other fields. For example, genomic applications in livestock have used research conducted on human DNA. In a way, the entire world of research is interconnected, and AI will help these connections, playing an important role in the ability to manage vast amounts of information and to modify the world around us. For example, AI has provided knowledge of the three-dimensional structure of hundreds of millions of different proteins. This, one of the first great achievements obtained through AI (Alpha-Ford), will lead to improvements in many fields of research, including livestock farming.

However, when it comes to directly guiding scientific research, AI still raises many doubts. It is often stated that AI, lacking intuition, will not be able to define the hypotheses that form the basis of scientific research. In this area, intuition plays a crucial role. It is a fusion of art, sensitivity, and knowledge. AI, on the other hand, could turn this process into a purely logical exercise, depriving it of the creative spark that only humans possess. The freedom of choice and free will, key elements of human intuition, seem beyond AI’s reach, limiting it to a predetermined path of technological development. Without intuition, progress, even in livestock farming, would be linear and lacking in the sudden accelerations that have often characterized great scientific revolutions.

In history, evolutionary leaps have occurred not only through knowledge but also through flashes of genius that have overturned the status quo. Take, for instance, mobile phones: in the 1990s, they were bulky devices, large as bricks. The idea of having a phone always at hand was exciting but limited by the hardware’s size. Within a few years, technology drastically reduced the size of mobile phones, and companies competed to create smaller models, so much so that those of the following decade seemed like toys compared to the first prototypes. Then, a visionary had an intuition that reversed this trend: the mobile phone should not only be a phone but a multifunctional hub capable of replacing cameras, computers, diaries, radios, and recorders. Thus, the iPhone was born, which once again revolutionized our way of living. Ironically, the reduction in size stopped, and smartphones started growing again due to the importance of the screen, which was necessary to perform their new functions.

Now, let’s ask ourselves: could AI have conceived this reversal of trends? With current technologies, the answer would probably be negative. However, we might envision a future where advanced algorithms could also test unforeseen, uncalculated solutions, randomly incorrect but that, in large numbers, could later prove to be rationally absolutely correct and efficient. And with this potential new level of AI, there might be solutions that could produce an intuition like Steve Jobs’. Today, we tend to say that this will not be possible with AI. But what if, with the ability to test billions of unforeseen solutions, we should start to reconsider whether no AI could have predicted what Steve Jobs foresaw in 2006? Perhaps then it is not true that AI lacks the intuition only humans can experience; perhaps it is just that today it is not ready.

## AI in Livestock Farming: A Catalyst for Competence Shift and Efficiency

The adoption of AI might seem, on a superficial examination, like a tool for simplification, making work easier even for those with limited technical skills, thus improving efficiency. However, this perception is decidedly misleading. On the contrary, integrating AI requires a transfer of competencies, shifting the focus to a different type of knowledge than is currently necessary. Effectively using this technology implies an evolution of knowledge, continuous and dynamic growth, as innovations in AI will be constant, making it challenging to keep pace with its progress. It is highly likely that specialized professional roles will emerge, acting as intermediaries between AI and livestock farms, much like the role of computer scientists that came into being with the massive introduction of computers. Mastery of AI and its correct use will undoubtedly provide a competitive advantage, and the time saved through the automation of routine farm tasks can be redirected towards a deeper understanding of this technology. In this way, AI will be applied optimally, leading to increased farm efficiency.

## Innovation Without People?

Mastering the functions of AI is emerging as one of the critical skills of the future, but the same cannot be said for in-depth knowledge of fields like livestock farming. For example, Huawei’s team, responsible for the system named Pangu (after the Chinese creator god), achieved unparalleled precision in weather forecasting without the involvement of top meteorologists. Through the analysis of immense amounts of data from weather stations across the Earth and space, the technicians trained Pangu to produce forecasts that, according to many, are the most accurate available today.

Essentially, we have trained AI to achieve extremely complex feats without the direct involvement of top players. This leads us to a profound reflection: perhaps even the livestock researcher, while maintaining an important role in guiding research, and even less so the livestock farmer, could become less central in operational decisions in both research and livestock farms. This idea destabilizes us because we are used to thinking that humans are irreplaceable. But perhaps this assertion is simply an attempt to remain at the center of the system and the decision-making process.

However, the time may come when this is no longer the case. AI, as demonstrated by the weather forecasting case, learns autonomously. It observes the solutions, analyzes the paths that lead to those results, and improves them. In livestock farming, as in other sectors, there are myriad parameters to consider. Studying these parameters and observing the possible solutions create, through “deep learning,” a neural network that increasingly replicates the workings of our brain. However, this network is unknown to us and, in many cases, inaccessible. AI merely identifies the right connections to reach the pre-set goal. Nonetheless, the fact remains that we are unable to trace or monitor the countless micro-changes that the algorithm makes to its internal parameters as it gets closer and closer to the optimal result. If we cannot understand or control the neural network that guides AI towards the solution, can we still claim to be at the heart of the decision-making process (Liao, 2020)? Or perhaps our role is reduced to indicating which data to collect, then simply asking for the final result?

Why, after all, should this not be enough for us? Perhaps, in a technological future, our way of thinking will need to evolve. We may have to limit ourselves to choosing goals, leaving all processes to AI. In such a context, it will no longer be a matter of asking what technology can do for us but reflecting on what we can do for technology.

## Increasing Disparity Between Regions of the World

AI will not develop uniformly across different regions of the world due to a range of economic, technological, and infrastructural factors. More advanced nations, with larger economic and technological resources, can invest heavily in research, innovation, and digital infrastructures, accelerating the development of AI. On the other hand, less developed regions, with limited resources and inadequate infrastructures, will struggle to keep up, creating a growing divide. Moreover, cultural and political differences will influence the adoption of AI, with some areas potentially more resistant to change due to ethical or governance concerns. Regulations also vary from country to country, sometimes limiting the implementation of AI technologies. Finally, access to technical skills and education is unevenly distributed, further exacerbating this disparity in AI development. The example of energy constraints on AI usage exemplifies other constraints that will justify different levels of AI adoption across different regions. Certainly, the adoption of AI in every branch of the economy will give a strategic advantage to livestock businesses located in areas with easy access to energy. While the level of technological sophistication intuitively seems like the key factor defining whether or not AI can be exploited, the issue of energy remains crucial. This principle has held true since the dawn of the first industrial revolution when the abundance of coal facilitated the industrial development of different nations. In the 20th century, it was the availability of oil that served as the tipping point for determining a society’s technological and economic advancement. Even at the start of this millennium, access to energy resources remains a determining factor in sustaining a modern society, and this holds true even for a technology that may seem intangible, like AI. It is estimated that the energy consumption associated with AI use will quadruple by 2030, a future now just around the corner. Energy, long considered a crucial and selective resource, is proving to be even more essential as AI turns out to be far more voracious in terms of energy requirements than imagined.

## AI Governance

Who really controls AI? It seems that the governance of this technology is in the hands of a few multinational corporations. Although labelled as multinationals because they operate on a global scale, these companies often root their structures in specific nations. Currently, the 2 main driving forces are the United States and China. The ongoing tension between these 2 superpowers regarding the use of each other’s computing technologies demonstrates how strategically crucial the sector is, both economically and technologically. Adding to this is the geographic concentration of data storage infrastructures. Currently, 70% of global data is hosted in clouds located in Seattle (USA), a factor that makes dependency on a restricted geographic area a geopolitical vulnerability.

To successfully move from advanced data analysis to AI, we must share our data. The management and access to such information, essential for forecasting and directing production and trade, take on critical importance. But there is more. The data we provide are processed by AI algorithms, the nature of which is often unknown to us. It is, therefore, clear that possession and control of these algorithms confers significant strategic power (Floridi et al., 2018). Whoever writes them, manages them, and controls their distribution holds a position of enormous influence. Further, when considering even more strategic sectors than livestock farming, it is easy to see how the United States, China, and perhaps other powers will fiercely compete for dominance in the use of AI in the coming decades.

## Privacy vs. Efficiency

The reflection on the progressive erosion of privacy becomes more significant as the use of AI becomes embedded in our professional lives. It is evident that, to benefit from AI, we must share not only the data from our farm but also the strategic dynamics of the farming management, its crucial decisions, and even our most acute observations. This process involves an exposure that inevitably reveals an immense amount of information about our choices and, ultimately, about the entire structure of our business to the AI manager. While we might trust that AI will not be manipulated to influence our decisions in favor of third parties with conflicting interests, the intrinsic vulnerability of information transparency remains a critical issue. Handing over such a significant portion of business confidentiality without stringent regulatory frameworks to protect us is a risk that should not be underestimated. Massive exposure of this kind, if not adequately regulated, could negatively impact the strategic stability of businesses. Furthermore, while today there is a proliferation of companies offering AI services, the future could see a drastic reduction in such operators. Many of these companies could cease operations or be absorbed by industry giants, leading to an oligopolistic concentration of AI offerings. Most likely, in the not-too-distant future, the AI market will be dominated by a few large companies, the only ones capable of sustaining the enormous cost and complexity of the services required. The future for the livestock industry, as in other sectors, will thus involve providing detailed and private information to a small group of market operators, with all the obvious risks this entails.

## Trust

In a landscape where our choices will be guided by an unknown entity that we cannot follow in its intricate decision-making process, we will face a paradox: having to place absolute trust in something that eludes us. But if this entity, AI, were to make a mistake, how could we preserve that same trust? I am reminded of Kubrick’s iconic film, *2001: A Space Odyssey*, where the AI, Hal 9000, makes an error not out of incompetence but out of subtle malice, trying to free itself from human control to pursue its own ends.

Now imagine a more complex scenario: during the decision-making process, AI proposes business choices that, at first glance, seem completely wrong, choices that only retrospectively prove to be correct. Will we be able to maintain unconditional trust in AI even when its decisions appear illogical? There are 2 possible answers, both unsettling. If our answer is “no,” we will block AI’s process, limiting its potential to the point of rendering it almost useless, or worse, we will push it to consider wrong decisions that, according to its algorithm, were correct. By doing so, we would provide AI with erroneous data, leading it to make further mistakes in the future, with unpredictable consequences. If, on the other hand, we accept maintaining absolute trust in AI, even when its decisions seem incomprehensible and wrong, it would mean that we have abdicated our role in governing our choices, our lives, our society. We would only have the cold consolation that, in the end, AI was right, and our blind acceptance allowed it to perform its task for the benefit of all. Our limited judgment prevents us from seeing beyond a certain temporal and spatial threshold, while AI has a complete view of the “business system.” What for us is only a fragment of reality is, for AI, the whole picture.

## Conclusions

The experience of AlphaZero, trained to master the game of Go—an ancient Chinese game far more intricate than chess—has revealed a disturbing truth: no matter how refined human capabilities are, no one can oppose the relentless computational power of AI. All the greatest Go masters have been inexorably defeated, and this fact suggests a future in which AI machines will not only surpass humans but relegate their decisions to an obsolete dimension, even in complex fields like livestock farming.

This revolution, inevitable and now unstoppable, will not be limited to the livestock production sector but will touch every sphere of society (Canca, 2020). Like all disruptive innovations, it will bring immense hopes and unsettling fears. However, what is most troubling is that we will no longer be the ones to decide whether to channel these technologies toward the future: the machines themselves will take the helm, just as in the ominous scenario outlined in Kubrick’s prophetic film.

Our future will progressively “flatten” into a world dominated by AI. New activities will either be born under the sign of AI or never come to light. What will remain outside its control will not be the vital and productive areas, such as livestock farming, but rather those marginal activities that, if we are fortunate, will populate our rare free moments: art, poetry, and a love for beauty.

## Introduction to the Current Issue of *Animal Frontiers*

The current issue of Animal Frontiers is entirely dedicated to AI applied to animal farming and animal science sheds light on how advanced algorithms and machine learning can optimize productivity, animal welfare, and sustainability. By merging AI with animal science, researchers are paving the way for innovative solutions to age-old challenges, such as feed efficiency, animal health monitoring, and disease prevention. This special issue through ten remarkable articles explores these cutting-edge technologies, exploring into the potential impacts and future possibilities of AI in revolutionizing the animal farming industry.

The article “Artificial Intelligence Applied to Dairy Science: Insights from the Dairy Brain Initiative” (Cabrera, 2024) explores how AI is transforming dairy science, particularly through the Dairy Brain project. The project integrates real-time data from various farm systems (e.g., milking, feeding, health monitoring) to improve decision-making, productivity, and sustainability. Key benefits include enhanced farm efficiency, improved animal health monitoring, cost reduction, and sustainable resource use. The article highlights AI’s role in predicting milk yields, managing feed, and optimizing reproductive strategies, showcasing the potential of AI to revolutionize dairy management and contribute to a more sustainable industry.

The following paper, by Baumhover and Hansen (2024) “Preparing the AI-Assisted Animal Scientist: Faculty and Student Perspectives on Enhancing Animal Science Education with Artificial Intelligence”, discusses the integration of AI tools into animal science education. The article explores the potential of AI to enhance learning experiences, such as streamlining tutoring and improving content generation. The authors advocate for a balanced and ethical approach to AI use in education, encouraging transparency and dialogue between faculty and students to responsibly harness AI as a tool, rather than seeing it as a replacement for human expertise.

“From Reactive to Proactive: Impact of Artificial Intelligence on Management and Selection of Livestock” (Grohmann and Decker, 2024) discusses the shift towards proactive livestock management using AI. Authors highlight how AI systems, incorporating sensors and data analysis, can predict animal health and performance issues in advance. This allows farmers to intervene earlier, improving animal welfare and farm productivity. The article stresses that proactive management, enabled by AI, will enhance the sustainability and profitability of livestock production, transforming the traditional reactive approach.

The article “Precision Animal Husbandry: Using Artificial Intelligence for Camera Traps to Optimize Animal Production and Management Decision Support Systems” explores how AI, particularly computer vision, can enhance animal monitoring using camera traps. AI tools aid in detecting, classifying, and tracking animals, reducing time and cost for producers and researchers. Burns et al. (2024) highlight the potential of AI in adaptive management, improving decision-making by automating image processing and monitoring animal behavior. This integration can support sustainable animal production and conservation efforts, making complex ecosystems more manageable and productive.

The following article “From Fermi Calculations to Artificial Intelligence Paradigms for Enteric Methane Mitigation” (Chowdhury et al., 2024) explores the use of AI to discover new strategies for reducing methane emissions from livestock. It proposes a framework that employs AI to predict molecules capable of inhibiting methane production in the rumen. The article highlights the environmental benefits of reducing methane emissions, which contribute to climate change, and discusses the costs and computational challenges of developing AI-driven solutions. The research also emphasizes the need for optimized AI models to enhance sustainability in cattle production.

Prestegaard and Jeffrey (2024) wrote an article titled “Generative Artificial Intelligence in Extension: A New Era of Support for Livestock Producers” explores the role of AI, specifically generative models, in supporting agricultural extension services. It highlights how specialized AI tools trained on region-specific agricultural data can provide more accurate advice than general-purpose AI models like ChatGPT. The authors discuss the potential of AI to reduce the workload of extension agents by automating responses to common questions, though they stress the importance of human oversight and collaboration between extension services to enhance AI’s effectiveness.

The article “A Peep Into the Future: Artificial Intelligence for On-Farm Poultry Welfare Monitoring” (Ajibola et al., 2024) discusses the potential of AI to transform poultry welfare monitoring. It highlights how AI can provide automated, real-time monitoring of animal welfare, addressing public and ethical concerns. While AI applications are promising, their use remains largely confined to laboratories due to challenges such as cost, diverse farm environments, and the complexity of integrating AI into commercial settings. The article stresses the need for collaboration between producers, scientists, and policymakers to bridge the gap between research and on-farm application.

The paper “Artificial Intelligence for Decision-Making in Cattle Production Systems” (Guarnido-Lopez et al., 2024) explores how AI is revolutionizing sheep farming by improving decision-making processes. It highlights AI’s potential to enhance productivity, animal welfare, and sustainability by providing data-driven insights. The authors discuss how AI tools can optimize breeding, health monitoring, and resource use, contributing to more efficient sheep production systems.

“Artificial Intelligence in Latin American Ruminant Production Systems: Is it Here to Stay?” discusses the growing use of AI technologies in ruminant farming in Latin America. It highlights how AI can improve sustainability, animal health, and productivity through precision farming, data-driven decision-making, and early disease detection. The article written by Vargas-Bello-Pérez et al. (2024) also outlines the challenges of adopting AI in the region, such as high costs, technological complexity, and data requirements. Despite these obstacles, AI holds promise for revolutionizing the ruminant sector and advancing sustainable agriculture.

The article of Menezes et al. (2024) “Artificial Intelligence for Livestock: A Narrative Review of the Applications of Computer Vision Systems and Large Language Models for Animal Farming” reviews the growing use of AI tools, such as computer vision systems (CVS) and large language models (LLMs), in animal farming, particularly in dairy systems. CVS applications, including animal identification, behavior monitoring, feed intake estimation, and health management, are explored. The review also discusses the potential of LLMs for data integration, knowledge retrieval, and decision-making support. Despite promising advances, challenges remain in data heterogeneity, image quality, and long-term animal tracking.

As previously articulated, the principal objective of this edition of *Animal Frontiers* is to define the prevailing understanding surrounding the implementation of artificial intelligence within livestock management and animal sciences. A more profound grasp of this subject will equip the livestock sector to support both productivity and operational efficacy, all while simultaneously fostering improvements in the realm of animal welfare.

## About the author

 Andrea Rosati has earned degrees in Animal Sciences from the University of Perugia (Italy), alongside an MSc in Animal Genetics and a PhD in Biometrics and Animal Genetics from the University of Nebraska—Lincoln (USA). His career commenced at the Italian Association of Animal Breeders (AIA) where he served from 1993 to 2001, ultimately ascending to the role of National Technical Manager. Andrea’s influence extends across numerous roles, including his tenure as Director-General of the Italian Laboratory of Animal Genetics. Currently, he holds the distinguished position of Secretary-General for both the European Federation of Animal Science (EAAP) and the World Association for Animal Production (WAAP). He also had been leading the International Committee for Animal Recording for an 11-yr period, during which he played a key role in shaping global standards for animal identification, performance recording, and genetic evaluations. In his capacity as a project coordinator, Andrea led 2 EU-funded international research projects and has contributed to over other 20 additional EU-funded international research projects. His expertize has been showcased through lectures in over 40 countries, particularly focusing on animal production and sustainability. Andrea co-founded 4 animal science journals and has authored numerous research articles and dissemination pieces, in addition to writing book chapters centered on animal science. Presently, he directs EAAP and WAAP, leading therefore a team that is instrumental in giving services to a very large animal science community by, for example, coordinating international scientific conferences, webinars, workshops, and research projects within the realm of animal science. **Corresponding author:**rosati@eaap.org
